# Patient-tailored transcranial direct current stimulation to improve stroke rehabilitation: study protocol of a randomized sham-controlled trial

**DOI:** 10.1186/s13063-023-07234-y

**Published:** 2023-03-23

**Authors:** Mia Kolmos, Mads Just Madsen, Marie Louise Liu, Anke Karabanov, Katrine Lyders Johansen, Axel Thielscher, Karen Gandrup, Henrik Lundell, Søren Fuglsang, Esben Thade, Hanne Christensen, Helle Klingenberg Iversen, Hartwig Roman Siebner, Christina Kruuse

**Affiliations:** 1 Neurovascular Research Unit (NVRU), Department of Neurology, Copenhagen University Hospital -Herlev and Gentofte, Copenhagen, Denmark; 2 Danish Research Centre for Magnetic Resonance (DRCMR), Centre for Functional and Diagnostic Imaging and Research, Copenhagen University Hospital Amager and Hvidovre, Copenhagen, Denmark; 3 Department of Nutrition, Exercise and Sport (NEXS), Copenhagen University, Copenhagen, Denmark; 4 Department of Physiotherapy and Occupational Therapy, Copenhagen University Hospital Herlev and Gentofte, Copenhagen, Denmark; 5 Department of Health Technology, Technical University of Denmark, Kgs Lyngby, Denmark; 6 Department of Radiology, Copenhagen University Hospital Herlev and Gentofte, Copenhagen, Denmark; 7 Department of Neurology, Copenhagen University Hospital Bispebjerg and Frederiksberg, Copenhagen, Denmark; 8 Department of Clinical Medicine, Faculty of Health and Medical Sciences, University of Copenhagen, Copenhagen, Denmark; 9 Department of Neurology, Copenhagen University Hospital, Rigshospitalet, Copenhagen, Denmark

**Keywords:** Transcranial direct current stimulation, Ischemic stroke, Neurorehabilitation, Upper-extremity recovery, Neuroplasticity

## Abstract

**Background:**

Many patients do not fully regain motor function after ischemic stroke. Transcranial direct current stimulation (TDCS) targeting the motor cortex may improve motor outcome as an add-on intervention to physical rehabilitation. However, beneficial effects on motor function vary largely among patients within and across TDCS trials. In addition to a large heterogeneity of study designs, this variability may be caused by the fact that TDCS was given as a one-size-fits-all protocol without accounting for anatomical differences between subjects. The efficacy and consistency of TDCS might be improved by a patient-tailored design that ensures precise targeting of a physiologically relevant area with an appropriate current strength.

**Methods:**

In a randomized, double-blinded, sham-controlled trial, patients with subacute ischemic stroke and residual upper-extremity paresis will receive two times 20 min of focal TDCS of ipsilesional primary motor hand area (M1-HAND) during supervised rehabilitation training three times weekly for 4 weeks. Anticipated 60 patients will be randomly assigned to active or sham TDCS of ipsilesional M1-HAND, using a central anode and four equidistant cathodes. The placement of the electrode grid on the scalp and current strength at each cathode will be personalized based on individual electrical field models to induce an electrical current of 0.2 V/m in the cortical target region resulting in current strengths between 1 and 4 mA. Primary endpoint will be the difference in change of Fugl-Meyer Assessment of Upper Extremity (FMA-UE) score between active TDCS and sham at the end of the intervention. Exploratory endpoints will include UE-FMA at 12 weeks. Effects of TDCS on motor network connectivity and interhemispheric inhibition will be assessed with functional MRI and transcranial magnetic stimulation.

**Discussion:**

The study will show the feasibility and test the efficacy of personalized, multi-electrode anodal TDCS of M1-HAND in patients with subacute stroke patients with upper-extremity paresis. Concurrent multimodal brain mapping will shed light into the mechanisms of action of therapeutic personalized TDCS of M1-HAND. Together, the results from this trial may inform future personalized TDCS studies in patients with focal neurological deficits after stroke.

**Supplementary Information:**

The online version contains supplementary material available at 10.1186/s13063-023-07234-y.

## Introduction

### Background and rationale

Ischemic stroke (IS) remains a global challenge and two-thirds of stroke patients show continued motor deficits which impact activities of daily living and quality of life [1]. Early-initiated rehabilitation training is central to recovery of motor function after IS [2, 3]. Transcranial brain stimulation (TBS) as an add-on to neurorehabilitation in the early subacute phase after IS (within the first 4 weeks after stroke onset) might result in faster and better recovery by optimizing the underlying neuroplastic processes, which may be more susceptible during the subacute phase post-stroke [4]. However, the use of a TBS technique to improve rehabilitation has to be feasible for patients and implementable in a clinical setting.

Transcranial direct current stimulation (TDCS) has been tested as a non-invasive tool to improve neurorehabilitation. The electric currents that TDCS can induce in the cortex through scalp electrodes result in a minor shift in the membrane potential and thereby a modification of the intrinsic neuronal network activity [5–7]. The pyramidal neurons in the area located under the anodal electrode are suggested to increase in excitability through depolarization of both the soma and the afferent axons while the pyramidal neurons located under the cathodal electrode decrease in excitability through hyperpolarization of the soma and afferent axons [8, 9]. These modulations in the membrane potential are thought to modulate behavior [10] and enhance neural plasticity by stimulating synaptic connections and long-term potentiation processes [11, 12].

Previous clinical trials have demonstrated that TDCS of the primary motor cortex (M1) may improve upper-extremity function in both subacute and chronic stroke patients, when applied concurrent with rehabilitation training. Ipsilesional anodal TDCS with the traditional montage of two square electrodes have been most widely examined [13–15], but contra-lesional cathodal TDCS [16, 17] and dual-TDCS [18] also has been studied. Across these studies, TDCS was applied to target the hand region of the primary motor cortex (M1-HAND) of either the healthy or the affected hemisphere, and current intensity is usually fixed between 1 and 2 mA across all subjects. However, according to meta-literature, up to 50% of patients are non-responders to the intervention [19], and only limited evidence of a significantly increased effect of TDCS compared to sham regarding upper-extremity rehabilitation [20–22]. Such lack of effects may associate to the *one-size-fits-all approach* which might miss the area thought to be targeted by TDCS (e.g., M1) both regarding location and current strength necessary to induce shifts in the membrane potential of the neurons in the target area. In addition, TDCS is often combined with either robot-assisted rehabilitation [22] or virtual reality [23] which may further confound the interpretation of the results.

Using individual magnetic resonance imaging (MRI) scans, electric field modeling enables a precise estimation of the electric field distribution in the brain during transcranial electrical brain stimulation and an optimization of electrode placement and dosing of interventional TDCS [24–27].

Patients with post-stroke upper extremity disability show an impaired motor network structure including a reduced excitatory influence from pre-motor brain areas and disinhibition of the contra-lesional M1-HAND [28, 29]. The interhemispheric imbalance between precentral motor cortices tends to improve with motor recovery and is often completely restored in patients with full recovery [28–30]. It is however unclear whether this imbalance facilitates or hinders motor recovery [29, 31].

### Objectives

The main hypothesis of this study is that patient-tailored anodal TDCS targeting the ipsilesional M1-HAND during supervised upper extremity training will result in greater improvements in upper-extremity function, measured by difference in change in FMA-UE score, compared to sham stimulation. It is furthermore hypothesized that patient-tailored TDCS is feasible to use for stroke rehabilitation in a stroke unit at a hospital setting.

Additionally, it is hypothesized that motor improvements correlate with the degree of normalization of functional motor connectivity and interhemispheric inhibitory interaction as revealed by task-related functional MRI and TMS. We will also assess the degree of corticospinal tract (CST) integrity measured by transcranial magnetic stimulation (TMS) and diffusion-weighted MRI (DWI) to explore how structural impairment in the corticospinal tract relates to the efficacy of anodal TDCS.

### Trial design

The study will be a parallel double-blinded two-arm randomized sham-controlled trial. In addition to usual care (preventive medication, advice on self-managed lifestyle changes, and municipal rehabilitation) the intervention group will receive two times 20 min of patient-tailored TDCS concurrent with supervised upper-extremity training for three times per week for 4 weeks in a 1:1 allocation ratio. The framework applied is exploratory.

## Methods: participants, interventions, and outcomes

### Study setting

Patients will be recruited during their admission at the Stroke Units of three participating University Hospitals of the Capital Region of Denmark (Region Hovedstaden): Copenhagen University Hospital Herlev and Gentofte, Copenhagen University Hospital Rigshospitalet, Copenhagen University Hospital Bispebjerg and Frederiksberg. Recruitment started in August 2022.

Patients will undergo routine clinical examinations for stroke patients and stroke subtype will be classified according to the Trial or Org 10,172 in Acute Stroke Treatment (TOAST) classification [32]. Patients will be grouped according to a cortical- or subcortically located stroke lesion for follow-up analyses that will explore the effect of stroke location on the potential beneficial effects of real TDCS.

See Table 1 for routine clinical examinations and demographic information collected.

**Table 1:** Routine examinations and demographic information

Routine examinations	Demographics
Blood samples Chest x-ray Magnetic resonance imaging of the brain Carotid ultrasound Computerized tomography (CT) angiography or MRI time of flight (TOF) of the brain to screen for intracranial stenosis Electrocardiogram (ECG) Holter monitoring for 72 h Transthoracical ecocardiography (TTE) in patients < 65 years	Medical history Prior and concurrent medication Smoking status (former, active never smoker) Alcohol consumption (weekly), Pre-morbid modified Rankin Score Education level, Pre-morbid walking status, Pre-morbid living arrangements Marital status, Stroke severity Active hand movement at stroke onset Ability to walk unassisted at stroke onset Administration of thrombolysis (IVT) or reperfusion therapy (EVT) prior to inclusion

### Eligibility criteria

Inclusion criteria for the patients are ischemic stroke lesion either located cortically or subcortically in the large hemispheres, symptoms presenting with any degree of arm paresis, age ≥ 18 years, able to speak and read Danish, and able to give informed consent and index stroke within 28 days of inclusion.

Exclusion criteria are > 50% stenosis of extra- or intracranial vessels, > 1 cerebral infarct or stroke event during admission, cerebral aneurysms or cerebral arterio-venous malformations, stroke location outside the large hemispheres, cognitive dysfunction interfering with the ability to participate, history of seizures, epilepsy or epilepsy in first-degree family, anxiety, dementia, alcohol- and drug abuse, headaches > 16 days per month or migraine as these can be provoked by TDCS and TMS, current use of neuro-receptor/transmitter modulating medication, medication reducing seizure threshold or prior adverse effect to TDCS or TMS, contraindications to MRI, or claustrophobia.

### Intervention — rehabilitation training

A training program designed to meet the individual needs and challenges of the patient will be planned in a pretraining session. Based on the evidence-based practice within neurorehabilitation the training will be goal-directed, repetitive, and task-specific [33, 34] with a focus on reaching, grip, and fine motor skills. See Supplemental Materials S1 for a detailed description of the exercise framework.

The participant will be encouraged to remain physically active. Furthermore, the patients will be instructed in two to four individual home-based exercises which they will do between intervention days. The number of repetitions and time spent on home-based exercises will be recorded in a pen-and-paper log by the patient in order to record compliance (see Supplemental Material S1).

There are no concomitant care or interventions (including medications) prohibited during the trial for both arms.

Intervention sessions will take place at Copenhagen University Hospital Herlev supervised by a trained occupational- or physiotherapist. Each training session will consist of two sessions of 20 min exercise with concurrent TDCS separated by a small break (≈5 min). The type of TDCS, active or sham TDCS, will be determined by randomization after inclusion of the patient.

The exercises will advance in difficulty gradually as the patient improves which is consistent with earlier findings that progressive practice improves motor skill learning and increased corticospinal plasticity [35, 36].

### Intervention — transcranial direct current stimulation

**Fig. 1:**
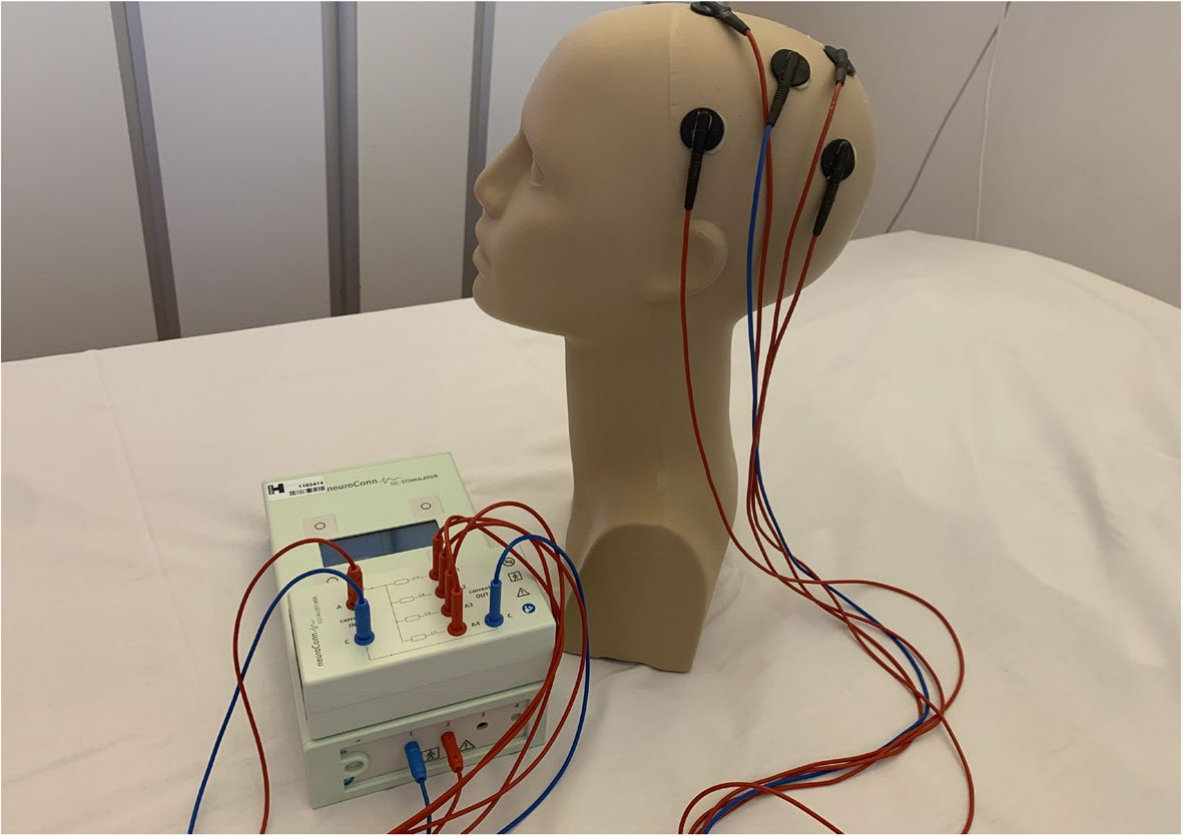
NeuroConn DC-STIMULATOR PLUS and an example of TDCS 1 + 4 round electrode montage

Focal anodal TDCs of the ipsilesional M1-HAND will be given via a multiple-electrode 4 + 1 montage using 20 mm round rubber electrodes (Richardson et al. [37], Alam et al. [38]) and a DC-STIMULATOR PLUS connected to a neuroConn Equalizer Box (NeuroConn, Ilmenau, Germany). Electrodes will be fixed on the scalp using Ten20® conductive paste in an approximately 0.2 cm thick even layer and covered by a net cap. The scalp will be prepped prior to electrode placement with NuPrep® skin scrub and alcohol swaps. The target electrode will be positioned over the ipsilesional hand knob area and the four cathodal return electrodes will be positioned equidistant surrounding like a ring with 60 mm to the target electrode (see Fig. 1).

**Fig. 2:**
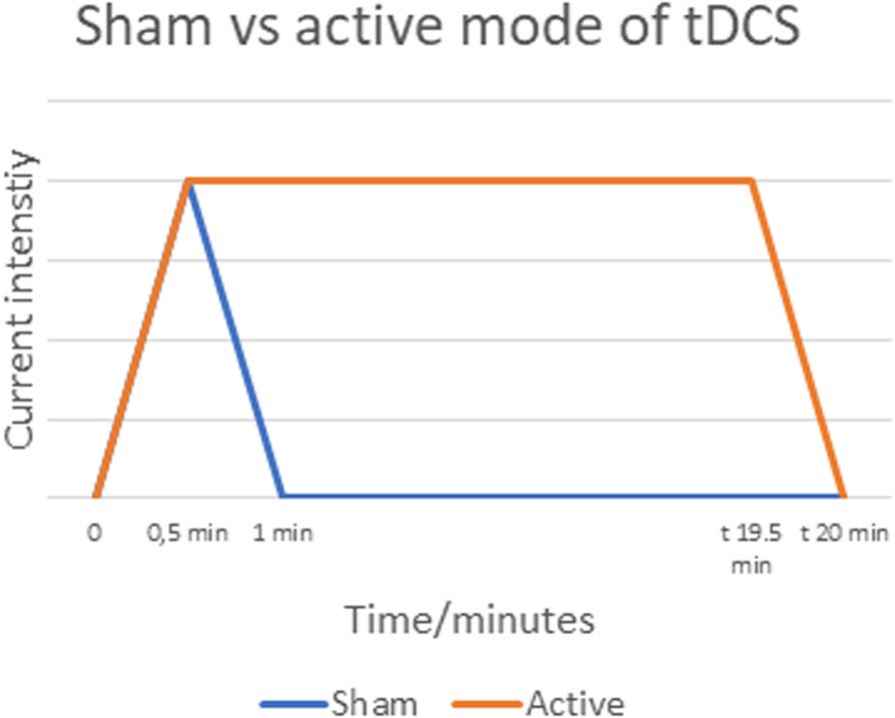
Diagram displaying the active and sham mode of transcranial electrical stimulation

Active stimulation mode consists of 30 s ramp followed by 19 min of stimulation and 30 s ramp-down to 0 mA. On the sham mode, there will be 30 s ramp-up and 30 s ramp-down [10, 39] to a small 50-μA sinusoidal current followed by 30 s ramp-down to 0 mA. See Fig. 2. The 50-μA current does not induce any physiological effects but allows impedance testing whereby the display at the DC stimulator appears identical in active and sham mode. The sham mode was preprogrammed in the DC-STIMULATOR at neuroConn Technology.

TDCS intensity will be calculated based on personalized electrical field models and adjusted to reach a field intensity of 0.2 V/m in the target area [40]. Both, the center electrode position, and current intensity will be individualized to each patient. We anticipate that personalized electrical field modeling will result in current intensities of 1.5–2.5 mA in most participants. TDCS intensity will not exceed a maximum current of 4.0 mA.

### Criteria for discontinuation

Patients not able to complete > 75% of the intervention sessions, patients with side effects from TDCS (such as severe headache following each intervention session), patients not able to complete the MRI scans, readmission with recurrent stroke or admission with another condition that contradicts TDCS, or upper-extremity exercise will be discontinued from the study.

### Strategies to improve adherence to intervention

Study retention will be promoted by a phone call 6 weeks after intervention has ended. The content of the training program will change weekly as the patient improves to promote motivation.

### Magnetic resonance imaging

At baseline, after the 4-week intervention and 12 weeks later whole-brain MRI scans will be acquired with a 3 T Philips Achieva scanner (Philips, Amsterdam, The Netherlands). The structural MRI protocol includes T1-weighted, T2-weighted, and fluid-attenuated inversion recovery (FLAIR) sequences and diffusion-weighted imaging (DWI). Functional brain mapping will include task-related blood oxygen level-dependent, functional MRI (BOLD-fMRI), and brain perfusion measurements with pulsed continuous arterial spin labeling (pcASL). Table 2 gives a detailed description of each sequence.

**Table 2:** MRI acquisition details

Sequence	T1W	T2W	FLAIR	fMRI BOLD	pcASL	DWI
Voxel size (mm)	0.85 × 0.85 × 0.85	0.85 × 0.85 × 0.85	1.0 × 1.0 × 1.0 mm	3.00 × 3.05 × 3.00	3.00 × 3.08 × 6.00	2.00 × 2.04 × 2.00
FoV (mm)	245 × 245 × 208	1245 × 245 × 190	256 × 256 × 202	192 × 192 × 126	240 × 240 × 126	224 × 224 × 100
TR (ms)	7000	2500	4800	2490	4607	7700
TE (ms)	3.3	265	314	30	18	64
Flip angle	8°	35°	40°	80°	90°	90°/180°
Acquisition time (min:sec)	05:40	06:32	05:40	05:06^a^	09:26	05:31 + 0:53
Readout method (EPI, FEE, TFE, TSE)	TFE 243	TSE 133	TSE 182	FFE/EPI	FFE/EPI	PGSE/EPI
SENSE/TSE/halfscan factor	SENSE 2 (AP)	SENSE 2 (AP), 1.8 (RL)	SENSE 1.8 (AP), 1.9 (RL)	n.a	SENSE 2.0 (AP)	Halfscan factor 0.8 SENSE 2 (AP)
Other			Inversion time: 1650 ms	Monitoring of respiration and pulse (PhysioLog)	Post-label delay: 2 s	b = 1000 s/mm^2^ (gradient ampl. = 62mT/m, duration = 12.5 ms, separation = 27.5 ms) 40 gradient directions and 6 interleaved b = 0 Additional 5 b = 0 scans in the AP-PA direction every five condition

^a^Sequence repeated three times

#### Task-related fMRI

Task-related fMRI will employ two runs featuring different manual motor tasks: The first fMRI paradigm records BOLD signal changes during a unimanual index-finger tapping task. Participants will produce irregular finger taps in response to a central visual cue at a pace of 0.5 Hz with a jitter of 0.25 s. The task will be performed in a single fMRI run, lasting 5 min. The second fMRI paradigm uses a block design to probe BOLD signal changes during a visually cued bimanual motor task. In the bimanual paradigm, participants have to generate bimanual responses with their index fingers in four visually cued conditions, “left before right finger,” “right before left finger,” “simultaneous index finger response,” or “no press” in a pseudorandomized order. Each task condition is presented in a pseudorandomized order in blocks of 20 s for a total of 5 min. In each condition, button presses will be triggered by a central visual cue at a pace of 0.5 Hz with a jitter of 0.25 s. The bimanual fMRI paradigm will be tested in two fMRI runs, lasting 5 min each. See Supplemental Figure S4 for an illustration of the visual cues.

All patients will be trained prior to each scan session to ensure an accurate and consistent performance of the motor tasks. Motor performance will be recorded with a four-button bimanual hand-held response pad (Cambridge Research System, Cambridge, UK). The visuomotor tasks are programmed with PsychoPy® [41] and visual cues were presented on an MRI-compatible screen behind the scanner on a mirror mounted on the head coil.

#### Arterial spin labeling (ASL)

Whole brain perfusion maps will be measured by pulsed-continuous ASL (pcASL). See Table 2 for sequence details. Post-label delay is set for 2 s as recommended in an elderly population [42]. Patients are instructed to relax and stay awake during the ASL. We will use pcASL to compare changes in whole brain perfusion from baseline to follow-up between the patients receiving active vs. sham TDCS, respectively.

#### Diffusion-weighted imaging (DWI)

DWI of the brain will be done to segment the CST and transcallosal motor fiber tract that connects the left and right M1-HAND and to characterize the microstructural damage of these motor white-matter tracts. The details of the MRI sequence used for DWI are described in Table 2. Using a diffusion tensor model, we will evaluate fractional anisotropy (FA) and mean diffusivity (MD) in the CST between the affected and unaffected hemispheres. Regional MD is acutely reduced after stroke but decreases to or below normal values in the weeks after infarct, reflecting a loss in cell density and tissue integrity [43–45]. Regional FA is a voxel-wise measure of the directionality of water diffusion and is sensitive to axonal alignment, density, and integrity [46]. A disruption of corticospinal tract integrity by a stroke lesion increases regional FA in the ipsilesional CST resulting in a FA asymmetry between the affected and the unaffected hemisphere [45, 46]. Tract segmentation will be performed with TractSeg [47] combined with custom MATLAB (MathWorks) scripts as a quantitative biomarker of microstructural white matter changes.

### Transcranial magnetic stimulation

Single- and paired-pulse TMS is performed at pre-interventional baseline and at either one or both follow-up visits to estimate the integrity of the CST as well as to evaluate corticomotor excitability. Specifically, the maximal amplitude of the motor evoked potential (MEP), cortico-motor conduction time (CMCT), contralateral silent period (cSP), ipsilateral silent period (iSP), and short intracortical inhibition (SICI) [48, 49] will be measured. TMS will be delivered with a hand-held figure-of-eight coil (MC-B70) connected to a MagPro 100 option stimulator (MagVenture, Farum, Denmark). The TMS evoked motor responses will be recorded with self-adhesive surface electrodes (Neuroline 700, Ambu, Ballerup, Denmark) attached to the left and right contralateral first dorsal interosseus (FDI) muscle using a belly-to-tendon montage. Electromyographic signals will be sampled at 5 kHz, band-pass filtered (5–2000 Hz) and amplified (1000), digitized, and stored using an eight-channel DC amplifier (1201 micro Mk-II unit, Digitimer, Cambridge Electronic Design) and Signal software version 4.11 (Cambridge Electronic Design, Cambridge, UK).

The cortical motor hotspot, the scalp position at which TMS produces the largest MEP, will be determined functionally and recorded using stereotactic neuronavigation (Localite, Bonn, Germany) throughout the experiment to ensure precise coil positioning. Resting motor threshold (RMT) will be determined as the stimulation intensity eliciting an MEP of > 50 mV in 5 out of 10 stimulations [50] and active motor threshold as an MEP of > 200 mV and a visible cortical silent period during a 10% maximal voluntary contraction (MVC) (if possible).

We will evaluate the excitability of intracortical GABAergic inhibitory circuits by measuring the strength of short intracortical inhibition (SICI) with paired-pulse TMS, applying a conditioning stimulus at an intensity of 80% RMT 2.1 ms prior to a test stimulus at an intensity of 120% RMT [51]. Twenty conditioned MEPs and 20 non-conditioned MEPs will be recorded to obtain reliable estimates of MEP amplitude for each stimulation condition.

Transcallosal inhibition will be estimated by recording the iSP. To this end, 20 pulses at 150% of RMT will be applied, while the patient performs a 50% MVC of the FDI muscle ipsilateral to the stimulated hemisphere. Lastly, maximum MEP amplitude, corticomotor MEP latency, and cSP will be determined using 20 TMS pulses at 150% RMT during 10% MVC of the contralateral hand. Moreover, we will determine the M-wave and F-wave latencies from 20 supramaximal constant current ulnar nerve stimulations (Digitimer DS7A, Cambridge, UK) to determine CMCT [48].

### Field modeling and electrode positioning

The scalp location of the TDCS center electrode and current intensity will be individualized using a SimNIBS pipeline based on the T1- and T2-weighted images of the individual patient [25, 40].

A custom SimNIBS script (SimNIBS version 4.0) will determine both the current intensity necessary to reach a mean field strength of 0.2 mV/m as well as locate the position of the target electrode on the scalp of the patient using a mask drawn in *fsaverage* for the hand knob area on either left or right hemisphere depending on location of the stroke lesion [52].

**Fig. 3:**
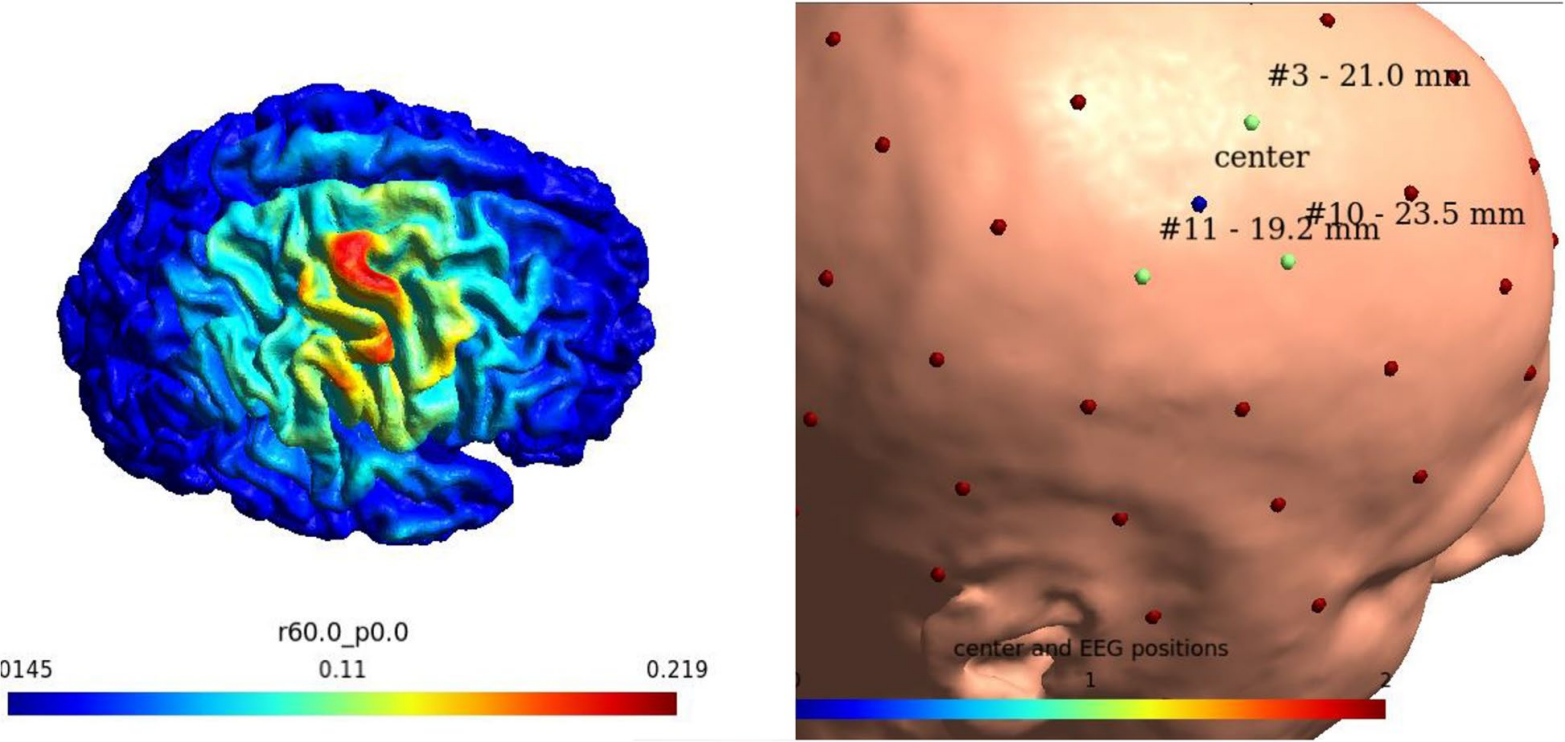
The use of SimNIBS to determine the current strength of TDCS necessary to reach a field strength of 0.2 mV/m in the target area as well as the distance from the target point to EEG positions on a head mesh to transfer the target position to a position of the scalp in real life

The position of the target electrode will be visualized on a 3D head mesh using a custom SimNIBS script displaying the three nearest EEG positions corresponding to a 64-channel EasyCap® M10-layout EEG-cap (EasyCap_BC_TMS64_X21, EasyCap, Woerthsee-Etterschlag, Germany). See Fig. 3. An individual EasyCap EEG-cap will be fitted for each patient prior to starting the intervention on which the position of the target electrode is marked with a hole. This is used to mark and ensure consistent electrode positioning during the intervention period. Surround electrodes will be positioned in 60 mm equally distributed as a ring around the target electrode. Five landmark positions will be used to ensure consistent fitting of the cap between sessions (Cz, Fpz, and Iz for frontal alignment and T8 and T7 for left–right-alignment).

### Primary outcome

The primary outcome is the difference in change of FMA-UE score at follow-up after 4 weeks of intervention compared to baseline between active TDCS vs. sham-stimulation.

The FMA probes five domains: motor function, sensation, balance, joint pain, and joint range of motion in the upper and lower extremities of hemiplegic stroke patients. It can be performed as either full-FMA or as upper or lower extremity FMA (FMA-UE or FMA-LE, respectively). In this study, FMA-UE will be assessed. Each of the five domains contains different items for assessment which are scored on a 3-point scale: 0 = cannot perform. 1 = performs partially, and 2 = performs fully. Impairment severity is based on FMA motor scores. Maximum score for FMA-UE is 66 points [53, 54].

### Secondary outcomes

#### Motor function and activity

Difference in improvement in upper-extremity function from baseline to follow-up in the active and sham-group will also be assessed with Action Reach Arm Test (ARAT). ARAT is composed of 19 items categorized into four subscales (grasp, grip, pinch, gross movements) arranged hierarchically with decreasing difficulty Task performance is rated on a 4-score scale ranging from 0 = “no movements” to 3 = “movements performed normally” [55]. Additionally, we will register the time spend on each task because the time frame resulting in the score of 2 (“task completed”) is large covering from 5 to 60 s. The Action Research Arm Test (ARAT) is currently being validated to the Danish language in a separate study by co-author KLJ (ID: H-20046644).

Changes in stroke severity and daily activity will be assessed by the National Health Institutes Stroke Scale (NIHSS) score, Modified Rankin Scale (mRS) [56], 20-item Barthels Index (BI-20) [57], 10-m-walk-test [58]. See also Table 3 as well as S3.

**Table 3:**
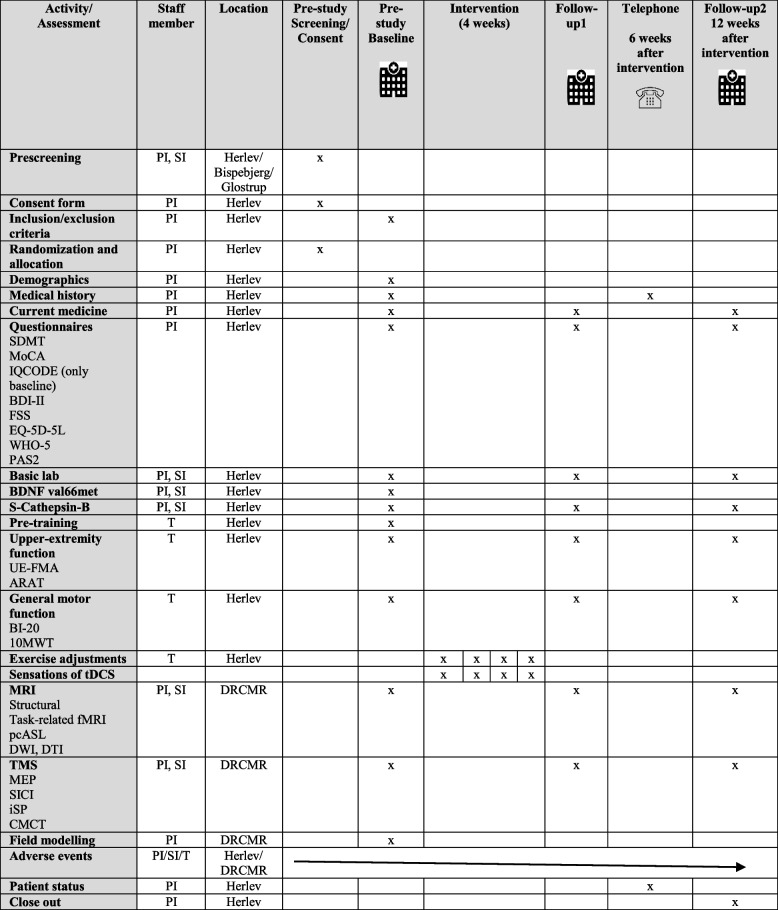
SPRIT-figure: schedule of study procedures

*SDMT* symbol digit modalities test, *MoCA* Montreal Cognitive Assessment, *IQCODE* Informant Questionnaire on Cognitive Decline in the Elderly, *BDI-II* Beck’s Depression Inventory II, *FSS* Fatigue Severity Scale, *WHO-5* World Health Organization – Five Well-being Index, *PAS2* Physical Activity Scale 2, *UE-FMA* Upper-extremity Fugl-Meyer Assessment, *ARAT* Action Reach Arm Test, *BI-20* Bartel-20 Index, *10MWT* 10-m walk-test, *MEP* motor evoked potential, *SICI* Short interval intracortical inhibition, *iSP* ipsilateral silent period, *CMCT* cortico-motor conduction time

#### Cognition

Baseline cognitive level will be evaluated by Informant Questionnaire on Cognitive Decline in the Elderly (IQCODE*)* [59, 60]. Cognitive changes will be assessed by Montreal Cognitive Assessment (MoCA) [61] and Symbol Digit Modalities Test (SDMT) [62]. See also Table 3 as well as S3.

#### Fatigue, mental well-being, and degree of depression

*Fatigue* will be examined by the Fatigue Severity Scale (FSS) [63]. *Mental well-being* will be evaluated by the EQ-5D-5L-test of health [64] and the World Health Organization – Five Well-Being Index (WHO-5) [65]. The degree of depression will be evaluated by Beck’s Depression Inventory-II (BDI-II) [66]. See also Table 3 as well as S3.

All tests and questionnaires used have been validated for stroke patients and are available in the Danish language.

#### Motor network connectivity and interhemispheric imbalance

Changes in the interactions between the motor network structures of the affected and unaffected hemispheres are important as they correlate with motor recovery. They will be assessed by effective connectivity during task-related fMRI. Using dynamic causal modeling (DCM) the direct influence (either facilitatory or inhibitory) of one region of interest (ROI) over another can be estimated (Friston [67]). The ROIs of this study are the primary motor cortex (M1), the supplementary motor area (SMA), and the ventral and dorsal premotor cortex (vPMC and dPMC, respectively) as these areas are involved in the motor network disturbances after stroke [68, 69]. Furthermore, the imbalance in the degree of activation of the motor cortex in the affected vs unaffected hemisphere will be determined by the laterality index (LI), as this is also correlated with motor recovery and tends to change to a more balanced activation with recovery [70].

#### Blood samples

A battery of routine blood samples (basic lab) will be collected at baseline and at each follow-up visit for safety and evaluation of cardiovascular risk factors (hemoglobin, hematocrit, leukocytes, C-reactive protein, blood platelets, international normalized ratio (INR), activated partial thromboplastin time (aPTT), fibrinogen, high-sensitive CRP (hsCRP) and apolipoprotein(a) (LP(a)). Additional blood will be collected at baseline for genetic analysis of brain-derived neurotrophic factor (BDNF) Val66Met genetic polymorphism, since this allele variant may influence the effect of TDCS [71] (De la Rosa 2019). Furthermore, blood for determination of plasma levels of cathepsin B will be collected at baseline and at each follow-up visit for measurements, as this lysosomal protein may relate to the cognitive effects of exercise [72]. See also Table 3 as well as S3.

### Participant timeline

All patients will be assessed at baseline directly after enrolment and at follow-up1 (after the 4 weeks of intervention has ended) and follow-up2 (12 weeks post-interventions).

**Fig. 4:**
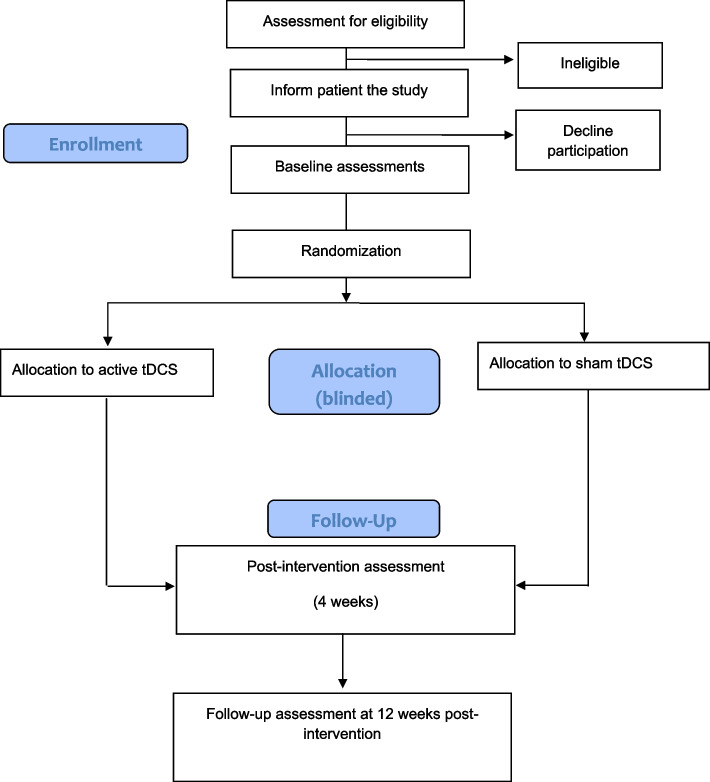
The PRACTISE-trial flow diagram

**Fig. 5:**
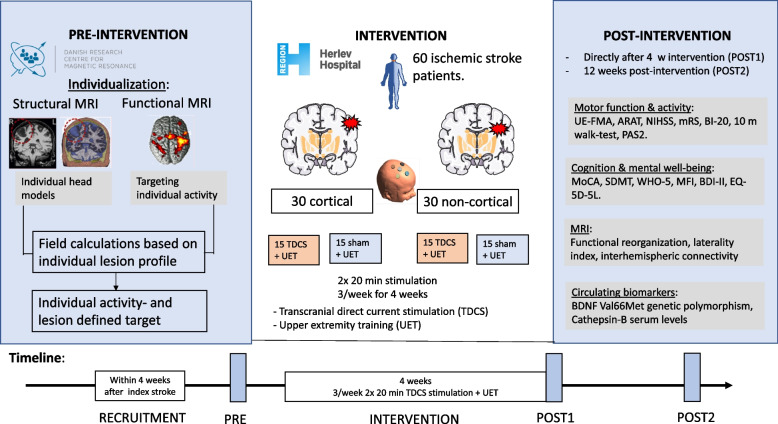
A graphical synopsis of the PRACTISE trial

See Table 3 for SPIRIT-figures of study procedures, Fig. 4 for a study flowchart, and Fig. 5 for a graphical synopsis of the study protocol.

### Sample size

The sample size is calculated based on the primary outcome. We expect an average difference in UE-FMA between groups of 5 points with a standard deviation of 3–4 points [23]. Assuming a Type I error of 0.05 and a Type II error of 80% 12 participants per arm are needed to reach sufficient power. To account for dropouts, we will include a minimum of 15 patients in each arm. An interim analysis will be done after the inclusion of 24 patients. To consider any differences due to the location of the stroke lesion we aim to include 30 patients with a cortical stroke lesion and 30 patients with a subcortical stroke lesion. However, if recruitment is more challenging than expected a feasibility analysis will be done after the inclusion of 30 stroke patients stratifying for infarct location (cortical vs. subcortical) in the analysis.

### Recruitment

Eligible patients will be identified by daily screening of medical records, and they will be provided with both oral and written information about the study. In case of severe aphasia, a next of kin participating in the information meeting may co-sign the consent if the patient wishes to participate in the study.

## Methods: assignment of intervention

### Allocation and sequence generation

Patients will be randomized consecutively into two groups, active or sham-stimulation, based on equal allocation. The allocation sequence is generated by SealedEnvelope™ (London, UK). Random-sized permuted blocks of participants will be applied at randomization. No stratification.

### Allocation concealment and implementation

At randomization, each patient is assigned a unique 5-digit code which will be typed into the DC-stimulator before each stimulation to apply either “active” or “sham” stimulation to maintain double blinding.

The randomization key containing the order of blocks and the allocation to either active or sham TDCS will be kept secured and unavailable to any delegates who are connected to the study until the trial is complete or in case an emergency unblinding is needed.

### Blinding

To remove any sensation of the TDCS the skin under the electrodes will be prepared with surface analgesics (Emla crème ®) 15 min prior to each stimulation. After each intervention session, the patient will be asked about any sensations from TDCS (such as skin itch, dizziness, headache).

At the last follow-up visit the patients will be asked: “Do you think you got active TDCS? Yes or no” for a quality assessment of the blinding. Furthermore, they will be asked: “Would you recommend TDCS and training for other stroke patients? Yes or no” for an assessment of feasibility.

## Methods: data collection, management, and analysis

### Data collection methods and management

All data recorded will be kept in the electronical report form (eCRF), REDCap™ (Vanderbilt University, TN, USA). All blood samples and lab results will be kept in the electronical medical file of the patient (see Fig. 1).

The collection of blood samples and the questionnaires regarding depression, quality of life fatigue, and activity level will be done by the study coordinator (MK). Assessment of motor function will be done by a study occupational- or physiotherapist that has not participated in the intervention sessions. All study therapists have received adequate training in all assessments of motor function prior to entering the study and will be supervised by an experienced coordinating therapist (KLJ). Clinical MRIs are described by an experienced neuroradiologist (KG). TMS data will be collected by co-investigators MJM and DH. MRI data will be collected by the study coordinator MK as well as sub-investigators MJM and DH. MJM is certified and experienced in TMS from several prior trials. MK has been certified in MRI and supervised by experienced senior researchers from DRCMR. MK has completed several pilot scans of both healthy and volunteering stroke patients independently prior to study initiation to ensure the quality of the MRI protocol. DH is certified in both TMS and MRI supervised MK and MJM and is experienced by several pilot sessions.

Checks for data entry errors and out-of-range errors are done in REDCap after each visit has been completed by each participant. Upper and lower limits are fixed for several data items in REDCap to ensure data entry within the normal range. After each TMS session inspection of the dataset for quality assessment and removal of outliers will be done. Furthermore, image quality is assessed during each MRI scan (e.g., appropriate field of view, movements, and other artifacts) and MRI data quality assessment will be done regularly along with regular testing of the MRI data analysis pipelines once a patient has completed both baseline MRI and follow-up visits. At the final follow-up visit missing data or any entry errors will be evaluated and handled.

### Statistical methods

All variables will be tested for normal distribution prior to analysis and logarithmically transformed if necessary. If data diverge from the normal distribution after transformation non-parametric testing will be performed. All tests will be two-sided and P < 0.05 will be considered significant. Data will be analyzed using Microsoft Excel 2010 (Microsoft Corporation, Redmond, WA, USA), *R* (version 3.6.1)*,* and REDCap or similar software. Statistical planning is conducted in a corporation with a biostatistician.

All data will be analyzed for the intention-to-treat population. All patients with complete outcome data will be analyzed according to the group they were randomized to. All available data for each patient will be included in the analysis. Missing data will be analyzed using imputation. Estimated treatment effects will be calculated based on a constrained longitudinal data analysis (cLDA) which will provide a result unbiased by values missing at random.

At follow-up 1 and follow-up 2 immediately after and 12 weeks after the intervention, respectively, a linear mixed model analysis will be used for both primary and secondary outcomes using cLDA. No other independent variables will be included in the analysis. The effect size will be calculated as mean change from baseline to follow-up and given as mean estimates of differences with a 95% confidence interval (CI). Patients that do not show up for a follow-up visit will be counted as missing values for the specific assessment point meant to be evaluated at that particular follow-up visit.

## Methods: monitoring

### Data monitoring

Confidential documents will be stored in a locked file, while the electronic information that can be traced to an identifiable person will be stored on a password-protected computer behind a secure “firewall” in accordance with the *Danish Privacy Act*. Data access will be limited to the study coordinator and sub-investigators involved in the study.

An interim analysis will be conducted after the inclusion of 24 patients.

### Adverse events monitoring and harms

Discomforts during the supervised training are expected to be muscle soreness and fatigue. All serious adverse events (SAEs) or side effects will be reported within 15 days to the sponsor and the Danish Medicines Agency (in Danish: *Lægemiddelstyrelsen*) and EUDAMED when implemented. A SAE is considered as an event resulting in considerable risk of or disability of the participant (or the offspring of the participant) including (but not limited by) death, permanent or severe disability/incapacity, hospitalization, or extension of hospitalization. All adverse events will be recorded in the electronic clinical report form.

### Plans for auditing and communication of amendments

Audits will be implemented on a yearly basis by the study sponsor. A protocol amendment was added for a 1-page patient information as a supplement for the detailed version of written patient information material (approved January 2021) and in October 2022 for repeating TMS at both follow-up visits after MRI as well as February 2023 for Bispebjerg Hospital as a recruitment site.

Any further protocol amendments will be communicated to ClinicalTrials.gov as well as collaborators.

The SPIRIT reporting guidelines were used for reporting the contents of this study protocol [73].

## Ethics and dissemination

### Research ethics approval

The study has been approved by the Research Ethics Committee in the Capitol Region of Denmark in November 2020 (H-20036199) according to the Declaration of Helsinki of 1964, revised in 2008 and approved by The Danish Data Protection Agency (ID: P-2020–921). The study is registered at ClinicalTrials.gov (ClinicalTrials.gov ID NCT05355831).

Selected elements of the study protocol have been tested in volunteering stroke patients prior to study initiation to ensure that the design is feasible for a patient population.

### Consent

Patients will be informed about the study and its contents by both oral and written informed consent obtained by the study coordinator (MK). The patient is given 24 h to consider participation. There is no post-trial care or any anticipated harm and compensation for trial participation.

### Confidentiality

To ensure confidentiality all patients are assigned to an identifiable study ID and names will never be included in the dataset.

### Dissemination policy

Results will be published in peer-reviewed international journals as well as be presented at national- and international conferences. Results will be published adhering to the CONSORT guidelines [74]. Co-authorship will comply with the Vancouver rules.

A letter will be sent to all participants explaining the results of the study in layman’s language.

## Discussion

TDCS has been used as an add-on treatment to exercise in several previous randomized controlled trials (RCTs) in stroke patients targeting motor deficits as well as language deficits (aphasia) or dysphagia [18, 20, 75, 76]. The clinical effects of TDCS on upper-extremity motor recovery in subacute stroke patients are inconsistent and up to 50% of the patients in the active group are non-responders. This suggests a need to examine the possible missing link between the application of TDCS and a clinical effect on the patient.

There is considerable heterogeneity in prior RCTs considering timing (before/during intervention), mode (anodal/cathodal) and duration of TDCS, current intensity, number of sessions, placement, shape, and size of electrodes as well as stage of stroke (acute, subacute, chronic) [20]. Few prior studies have investigated the use of the more focal HD-TDCS with a 1 + 4 electrode montage in stroke patients with aphasia [37, 77] and motor function in chronic stroke [78, 79]. Prior studies have used field modeling to simulate the optimal TDCS electrode placement but in chronic stroke patients [27, 80] and these studies did not individualize the current intensity of the stimulation. The largest recovery occurs within the first 12 months post-stroke. However, there is a “window of opportunity” within the first 30 days of the stroke onset in which neuroplasticity and thereby the potential for recovery is enhanced and the changes dramatic [2, 4]. It would therefore be appropriate to add TDCS already during this early phase of rehabilitation to faster achieve a better outcome which would allow the patient a faster return to a normal life with as minimal deficits as possible.

When searching MEDLINE, Scopus, and ClinicalTrials.gov there are no studies that combine anodal focal TDCS and field modeling to individualize treatment in a cohort of subacute stroke patients regarding upper-extremity motor function.

We suggest that this individualization is necessary to reach the full potential of TDCS as an add-on treatment for stroke rehabilitation in order to target clinically relevant areas for stimulation and use an appropriate current strength.

This study addresses these issues by individualizing TDCS for each subacute stroke patient using state-or-the-art MRI and field modeling techniques regarding both (1) the individual location of the ipsilesional hand knob area and (2) individual current strength to ensure a physiologically effective electric field distribution in the target area.

This study will further investigate the feasibility of patient-tailored TDCS for stroke rehabilitation in the daily clinical routine of a hospital stroke unit and if it is favorable to apply for the patient concurrent with rehabilitation. This is done both by drop-out rates, by tracking sensations during TDCS, and by asking the patient whether they would recommend TDCS for future stroke patients. We hope this study will also help clarify the process of upper-extremity motor recovery and the role of interhemispheric competition during this process.

In addition, it would be highly interesting to repeat the baseline measurements in a cohort of healthy age-matched individuals to compare the motor network organization and connectivity with stroke patients both in the subacute phase and in the later stages of motor recovery.

## Trial status

The current version of the protocol was approved by the ethics committee in November 2020. Recruitment at Herlev Hospital began in August 2022, recruitment at Rigshospitalet-Glostrup began in October 2022, and at Bispebjerg Hospital in February 2023. In February 2024 an interim study analysis of the first 30 included patients is expected to be conducted. Recruitment of patients is expected to be completed in late 2024.

## Supplementary Information

**Additional file 1.** Standard Operating procedures – Upper-extremity training.

**Additional file 2.** Exercise log – translated version and the original Danish version.

**Additional file 3.** Outcome measures, abbreviations, description and purpose.

**Additional file 4:** **Figure S4.** Visual cues for the fMRI paradigms.

## Data Availability

Data from MRI and TMS, clinical outcome data as well as will be made available upon request. A research biobank containing plasma, serum, and full blood from each patient from both baseline and follow-up visits stored at − 80 °C has been approved by the data protection agency and will be established. Data from the medical files of the participants will remain unavailable.

## Abbreviations

IS: Ischemic stroke
TBS: Transcranial brain stimulation
TDCS: Transcranial direct current stimulation
M1: Primary motor cortex
MRI: Magnetic resonance imaging
UE: Upper extremity
FMA: Fugl-Meyer Assessment
TMS: Transcranial magnetic stimulation
CST: Cortico-spinal tract
DWI: Diffusion-weighted imaging
BOLD-fMRI: Blood oxygen level-dependent, functional MRI
pcASL: Pulsed continuous arterial spin labeling
FA: Fractional anisometry
MD: Mean diffusivity
MEP: Motor evoked potential
CMCT: Cortico-motor conduction time
iSP: Ipsilateral silent period
SICI: Short intracortical inhibition
RMT: Resting motor threshold
AMT: Active motor threshold
ARAT: Action Reach Arm Test
NIHSS: National Health Institutes Stroke Scale
mRS: Modified Rankin scale
BI-20: 20-Item Barthels Index
10MWT: 10-Meter-walk-test
IQCODE: Informant Questionnaire on Cognitive Decline in the Elderly
MoCA: Montreal Cognitive Assessment
SDMT: Symbol Digit Modalities Test
FSS: Fatigue Severity Scale
WHO-5: World Health Organization – Five Well-Being Index
BDI-II: Becks Depression Inventory-II
DCM: Dynamic causal modeling
ROI: Region of interest
SMA: Supplementary motor area
vPMC: Ventral premotor cortex
dPMC: Dorsal premotor cortex
LI: Laterality index
INR: International normalized ratio
aPPT: Activated partial thromboplastin time
CRP: C-reactive protein
hs-CPR: High-sensitive CRP
LP(a): Apolipoprotein(a)
BDNF: Brain-derived neutrotrophic factor
CTSB: Cathepsin B
cLDA: Constrained longitudinal data analysis
SAE: Serious adverse event
RCT: Randomized controlled trial
