# Supplementary material for: Patient-tailored transcranial direct current stimulation to improve stroke rehabilitation: study protocol of a randomized sham-controlled trial
Source: Trials. 2023 Mar 23;24:216. doi: 10.1186/s13063-023-07234-y (PMC10035265; doi:10.1186/s13063-023-07234-y)

# Supplemental material 1

## Standard Operating procedures – Upper-extremity training

**Framework**

Based on the evidence-based practice within neuro rehabilitation the physical rehabilitation applied in the study will be goal directed, repetitive and task-specific (Nielsen 2015, Hubbard 2009, Winstein 2015) and consist of exercises for the supervised training and exercises for home-based training.

The upper-extremity (UE) training will be individualized and focus on reaching, grip, grasp and fine motor skills. It will accommodate the impairments of the participants and their goals for the training. The interventions (tasks) will be organized at an appropriately challenging level for each participant and use the full potential of the UE function regardless of the UE functional level to maintain motivation.

To plan the UE intervention each participant will undergo a movement analysis, where the therapist observes motor performance during different tasks identifying missing or decreased essential components compared to the normal movement pattern for the task. The tasks will be selected based on what the participant indicates as the main problems e.g., general activation of the impaired UE due to severe paresis, button a shirt, brush teeth, doing laundry etc. Hypotheses about which underlying impairments that might be causing the observed movement deficits will be investigated, this could be decreased range of motion, strength, coordination etc. The participants UE function will be divided into five levels which directs general guidelines for the exercises (Table 1). Based on these guidelines the therapist will choose different exercises for the supervised training.

*Grading and modification of the exercises*

During the supervised training, the exercises are continuously adapted and graded regarding level of difficulty (physical and cognitive), number of exercises and repetitions. The home-based exercises are adjusted once a week based on the participants goal, motor performance and exercise log.

For instance, if the participant completes the exercises without making any mistakes such as mishandling or dropping the object, the level of difficulty will be increased – e.g., by using smaller objects such as toothpicks instead of straws in fine motor exercises or using more weight e.g., add more water in a cop or pour water from a jug. Change in speed and coordination with the same demand of doing the exercise accurately can also increase difficulty of the exercise.

**Training sessions**

*Pre-training session*

Each participant will participate in a pre-training session scheduled prior to the start of the supervised training with a duration of approximately 45 minutes. In the pre-training session, the participant and the therapist discuss limitations in everyday activities due to upper limb (UE) impairments and the participants main goal/goals for the four weeks intervention. A movement analysis is performed followed by an examination of the underlying impairments. Goal and intervention for the first supervised training week is planed with the patient and expectations for the home-based exercises are clarified e.g number of exercises and number of repetitions or amount of time for each exercise.

*Supervised training*

The supervised training will occur three times per week for four weeks at Herlev Gentofte Hospital and consist of two sets of 20-minute training blocks with concurrent TDCS and a short break between training blocks. The duration of each training session is approximately 60 minutes including the break.

**Home-based exercises**

The home-based exercises comprise two to four exercises performed on a daily basis with a total duration of approximately 30 minutes. The home-based exercises are chosen so the participant can perform them independently, with high quality and as many repetitions as possible.

The participants receive an exercise log (see supplemental material S2) together with a description of the exercises (See figure 1S). In the exercise log the participant notes the number of repetitions and time spent on each exercise. The exercises are adjusted once a week.

In addition to the exercises the participants are encouraged to use the impaired UE as much as possible.

Table 1_Overview: Level of upper limb (UL) function and general guidelines for UL exercises.

|  | **Level 1** | **Level 2** | **Level 3** | **Level 4** | **Level 5** |
| --- | --- | --- | --- | --- | --- |
| **UL function** | Muscle activation  -Initiate muscle contraction over minimum one joint  Range of motion  Limited active range of motion with eliminated gravity, needs some support to have some active range of motion within the midrange.  Endurance  Severely affected, compound movements are limited.  The timing and coordination of movement  not possible | Muscle activation  -Muscle activation of ≥ 2 joints  - severely affected  hand function  - Minimal fine motor skills/dexterity  Range of motion  Limited active range of motion against gravity with the need for some degree of support to lift against gravity within the midrange  Endurance  Severely affected when movements are compound  The timing and coordination of movement  severely affected | Muscle activation  - muscle activation of shoulder, elbow, forearm, wrist and fingers.  - moderate affected strength  - Grasp and release with forearm supported is possible, but limited  - Fine motor skills moderately impaired  Range of motion  active range of motion is full when supported against gravity. Lift against gravity unsupported in the midrange is possible  Endurance  Moderate affected when movements are compound    The timing and coordination of movement moderately affected | Muscle activation  - muscle activation of shoulder, elbow, forearm, wrist and fingers  -mild affected strength  - able to grasp and release objects  - Fine motor skills mild impaired – able to manipulate objects  Range of motion:  Active range of motion is full against gravity. Lift against gravity unsupported in end range is possible. Lifting minor objects e.g. spice jar against gravity is possible.  Endurance:  Mild affected when movements are compound  The timing and coordination of movement  mild affected | Muscle activation  - normal muscle activation with normal strength and no side differences  - Normal fine motor skills  Range of motion:  Full against gravity  Endurance:  Normal – perhaps affected due to fatigue cognitive and/or physical  The timing and coordination of movement  normal |
| **General guidelines for UL exercises** | Practice of gross motor skills | Practice of gross motor skills and introduction of fine motor skills | Practice of gross motor skills and fine motor skills | Practice of gross motor skills and substantial fine motor skills primarily focusing on endurance, timing and coordination in exercise  transferable to activities of daily living | Focus on exercise  transferable to activities of daily living in combination with cognitive and/or physical demands during the exercises |
|  | -Part practice with eliminated gravity and within midrange  -Decreased friction e.g. use of towels or skateboard  -No involvement of the trunk during reaching/the exercises  - Dexterity/manipulation/ fine motor skills not possible. | -Part practice with eliminated gravity in the possible active range of motion  -Part practice against gravity with sustained contractions or with decreased friction  -minimal involvement of the trunk during reaching/exercises  - Dexterity/manipulation/ fine motor skills are limited.  -UL exercises performed within base of support | -Whole and part practice against gravity in the possible active range of motion  -Reaching and dexterity/manipulation is possible  - involvement of truncus when reaching  -UL exercises performed within and outside the base of support  -In some degree use of resistance in exercises e.g. theraband, grip dynamometer or weights  -Increase speed and timing | -Whole and part practice against gravity in full ROM  -Reaching and dexterity/manipulation is slightly limited  - involvement of truncus when reaching  -UL exercises performed within and outside the base of support  -Use of resistance in exercises e.g. theraband, grip dynamometer or weights  -Increase speed and timing | -Whole practice  -Strength and dexterity in combination with cognitive and physical demands e.g. UE exercise performed in standing outside the base of support and with different environmental influence  - involvement of truncus when reaching |
| **Goal setting,**  **examples** | -active shoulder shrug | -active arm involvement during dressing  - scratch the nose | - manipulation of a knife, fork and spoon  - Comb hair  - Shave  - Put toothpaste on the toothbrush  -get dressed  -pour water into a glass | - Texting messages  - writing  - button a shirt  - manipulation of a knife, fork and spoon | - Do laundry  - Do the dishes  - Empty the dishwasher  - Cook  - |
| **Exercises during tDCS** | Part practice | Part practice | Part practice  Whole practice | Part practice  Whole practice | Whole practice  Whole practice |
| **Example UL function** | Patients with severely affect UL, who can initiate activation e.g. over shoulder in elevation/ flexion/extension.  Activation is often seen in combination with a compensating strategy using the trunk to get a larger range of motion or by activating “all muscles” in order to try to make e.g. a grip.  Some patients can only initiate contractions over wrist and fingers without being able to initiate contraction over the shoulder and elbow.  Not possible to use the UL in ADL without guiding /help from another person. | Patients can perform some of the transport phase and can start to initiate voluntary activation over wrist and fingers. During exercises grip function needs to be supported.  Activation can be seen in combination with a compensating strategy using the trunk to get a larger range of motion or by activating “all muscles” in order to try to make e.g. a grip.  Possible to use the UL with a supporting function during ADL. | Patients can do exercises involving grip and graps and some fine motor skills.  Compensation is rare and often first seen when the patient gets tired.  Active involvement of affect UL during ADL in bimanual task. Need for some support of affected UL during ADL e.g. taking af t’shirt on or/and putting socks on. | Patients can integrate UL in ADL without any form of compensation. Patients especially experience limitation regarding endurance, timing and coordination. This gets more pronounced when the patients get cognitive and/or physical tied or when the patients get distracted. | Patients can integrate UL in ADL and describe UL as “being back to normal or almost back to normal”. Patients can experience limitation due to fatigue or with increasing cognitive and/or physical demands, where the activity is lightly affected when focus from the specific task is removed. |

**Fig. 1S. Example of a home-based exercise**


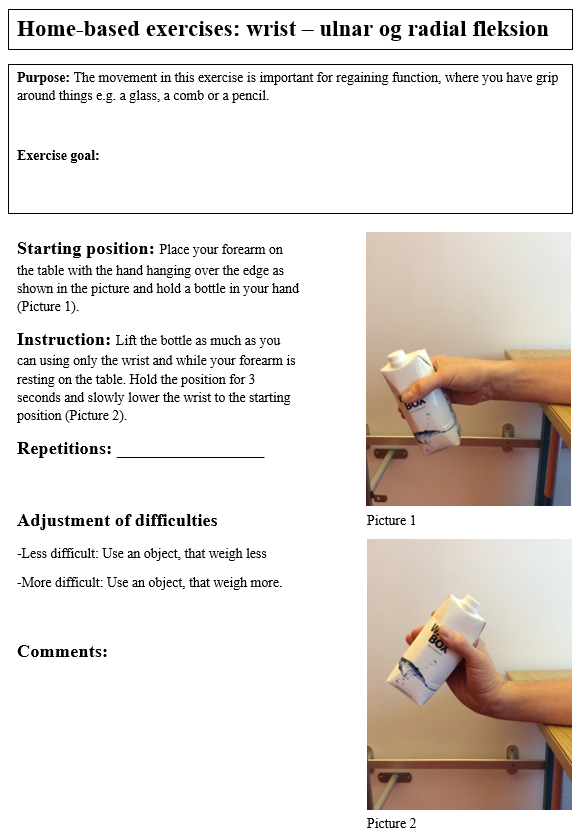

Supplement: Supplementary file 1 — Additional file 1. Standard Operating procedures – Upper-extremity training. [file 13063_2023_7234_MOESM1_ESM.docx]
