# Supplementary material for: Patient-tailored transcranial direct current stimulation to improve stroke rehabilitation: study protocol of a randomized sham-controlled trial
Source: Trials. 2023 Mar 23;24:216. doi: 10.1186/s13063-023-07234-y (PMC10035265; doi:10.1186/s13063-023-07234-y)
Supplement: Supplementary file 2 — Additional file 2. Exercise log – translated version and the original Danish version. [file 13063_2023_7234_MOESM2_ESM.docx]

S2. Exercise log – translated version and the original Danish version

**Week number:**

|  |  | **Monday** | **Tuesday** | **Wednesday** | **Thursday** | **Friday** | **Saturday** | **Sunday** |
| --- | --- | --- | --- | --- | --- | --- | --- | --- |
| 1: | Number of repetitions |  |  |  |  |  |  |  |
|  | Time spend |  |  |  |  |  |  |  |
| 2: | Number of repetitions |  |  |  |  |  |  |  |
|  | Time spend |  |  |  |  |  |  |  |
| 3: | Number of repetitions |  |  |  |  |  |  |  |
|  | Time spend |  |  |  |  |  |  |  |
| 4: | Number of repetitions |  |  |  |  |  |  |  |
|  | Time spend |  |  |  |  |  |  |  |
| **Comments:** | | | | | | | | |

**Ugenummer:**

|  |  | **Mandag** | **Tirsdag** | **Onsdag** | **Torsdag** | **Fredag** | **Lørdag** | **Søndag** |
| --- | --- | --- | --- | --- | --- | --- | --- | --- |
| 1: | Antal gentagelser |  |  |  |  |  |  |  |
|  | Tid |  |  |  |  |  |  |  |
| 2: | Antal gentagelser |  |  |  |  |  |  |  |
|  | Tid |  |  |  |  |  |  |  |
| 3: | Antal gentagelser |  |  |  |  |  |  |  |
|  | Tid |  |  |  |  |  |  |  |
| 4: | Antal gentagelser |  |  |  |  |  |  |  |
|  | Tid |  |  |  |  |  |  |  |
| **Kommentar** | | | | | | | | |
