# Supplementary material for: Patient-tailored transcranial direct current stimulation to improve stroke rehabilitation: study protocol of a randomized sham-controlled trial
Source: Trials. 2023 Mar 23;24:216. doi: 10.1186/s13063-023-07234-y (PMC10035265; doi:10.1186/s13063-023-07234-y)
Supplement: Supplementary file 3 — Additional file 3. Outcome measures, abbreviations, description and purpose. [file 13063_2023_7234_MOESM3_ESM.docx]

**Supplemental material S3**

**Outcome measures, abbreviations, description and purpose**

| **Outcome** | **Abbreviation** | **Description and purpose** |
| --- | --- | --- |
| **Primary outcome** | | |
| Upper-extremity Fugl-Meyer Assessment | UE-FMA | Assessment of recovery of upper extremity strenght by Δchange in UE-FMA score between the active and the sham stimulation group. |
| **Secondary outcomes** | | |
| Action Reach Arm Test | ARAT | Assessment of recovery upper-extremity mobility by Δchange in ARAT score between the active and sham stimulation group. |
| Bartels 20-Index | BI-20 | Assessment of general functioning in the daily living (feeding, bathing, grooming, dressing, toilet use, transfer and mobility) (Shah et al 1989) |
| 10-meter-walk-test | 10MWT | Assessment of general motor functioning, a measure not isolated to upper-extremity functioning. |
| Structural Magnetic Resonance Imaging | MRI | For brain segmentation in *fsaverage* for field modelling in SimNIBS.  Assessment of infarct lesion location, degree of cerebral small vessel disease, factional anisometry (FA) asymmetry and degree of lesion in the corticospinal tract. |
| Functional Magnetic Resonance Imaging | fMRI | Assessment of motor network changes during recovery measured by functional connectivity and degree of inter-hemispheric imbalance measured by changes in laterality index. |
| **Essential co-variates** | | |
| Stroke severity | | |
| Stroke severity measured by National Institutes of Health Stroke Scale | NIHSS | Assessment of stroke severity on 11 items: level of consciousness, horizontal eye movements, visual fields, facial palsy, motor arm, motor leg, limb ataxia, sensory, language, speech, extinction/inattention. A score of 0 indicates “no stroke symptoms”, a score of 1-4 indicates “minor stroke”, a score of 5-15 indicates “moderate stroke”, 16-20 “moderate to severe stroke” and 21-42 “severe stroke” |
| Modified Rankin Score | mRS | Assessment of degree of disability both before index stroke, at baseline and at follow-up. Furthermore, it is used as one of the inclusion criteria as only pt with mRS ≤3 will be included (Farrell et al 1991). |
| Cognition | | |
| Informant Questionnaire on Cognitive Decline in Elderly | IQCODE | Assessment of baseline difference in cognitive status between patients. IQCODE is a questionnaire for evaluation of cognitive decline containing 26 everyday situations in which memory, cognition or language ability is needed over the last 10 years (Jorm 1988). |
| Cognition measured by Montreal Cognitive Assessment and | MoCA | Assessment of seven different dimensions of cognitive function: attention and concentration, executive functions, memory, language, visuospatial skills, conceptual thinking, calculations and orientation (Nasreddine 2005). |
| Symbol Digit Modalities Test | SDMT | Assessment of attention, speed of processing, visual scanning, working memory along with visuospatial coordination and manual dexterity (Koh et al. 2011). |
| Depression, fatigue, activity level | | |
| Mental well-being | EQ-5D-5L | For description and evaluation of decrements in health in five dimensions: *mobility, self-care, usual activities, pain/discomfort, anxiety/depression* (Herdman et al 2011). |
| Depression measured by Becks Depression Inventory-II | BDI-II | Assessment of self-reported symptoms of depression examining several items such as hopelessness or irritability, cognitive symptoms, such as guilt or being disappointed with oneself, as well as physical symptoms, such as fatigue, weight loss and loss of interest (Turner 2012). |
| World Health Organization – Five Well-Being Index | WHO-5 | Assessment of the the mental well-being and consists of five positive statements to which the respondent must answer to what degree they agree on a 5-point Likert scale. The total score ranges from 20 to 100 - the higher score, the higher quality of life. A score of 50 or below is an indication for testing for depression or a sign of chronic stress (Awata et al. 2007). |
| Fatigue severity scale | FSS | Assessment of fatigue on a 9-item scale which determines degree of fatigue and how it influences daily activities (Ozyemisci-Taskiran et al. 2019) |
| Physical activity scale version 2 | PAS2 | Assessment of physical activity as daily hours and minutes of sleep, sitting, standing or walking as well as activity at work and transportation to and from work. In addition, PAS2 measures weekly hours and minutes of light-, moderate- and vigorous intensity activity for at total measurement of Metabolic Equivalent of Task (METs) spend per day. |
| Transcranial magnetic stimulation | | |
| Transcranial Magnetic Stimulation  Motor Evoked Potential, intracortical Silent Period, Short Intra-Cortical Inhibition, Cortico-Motor Conduction Time. | TMS, MEP, iSP, SICI, CMCT | Evaluation of corticospinal excitation and interhemispheric inhibition as well as conduction time compared at baseline vs. after four weeks of intervention and 12 weeks after intervention . |
| General lab and biomarkers | | |
| General lab  (Hemoglobin, hematocrit, leukocytes, C-reactive protein, blood platelets, international normalized ratio, activated partial thromboplastin time, fibrinogen, high sensitive CRP and apolipoprotein A) | Hgb, hct, lkc, CRP, trbc, INR, aPTT, hsCRP, LP(a) | For safety and evaluation of coagulation and cardiovascular risk factors |
| Brain-Derived Neurotrophic Factor genetic polymorphism | BDNF Val66Met | Evaluation of frequency of the Val66Met allelle in the study population and if this has an impact on motor recovery and effect of tDCS. |
| Cathepsin B | CTSB | Assessment of changes of plasma-level CTSB and if this has an impact on effect of TDCS. |
