# Supplementary figures and images for: Patient-tailored transcranial direct current stimulation to improve stroke rehabilitation: study protocol of a randomized sham-controlled trial
Source: Trials. 2023 Mar 23;24:216. doi: 10.1186/s13063-023-07234-y (PMC10035265; doi:10.1186/s13063-023-07234-y)

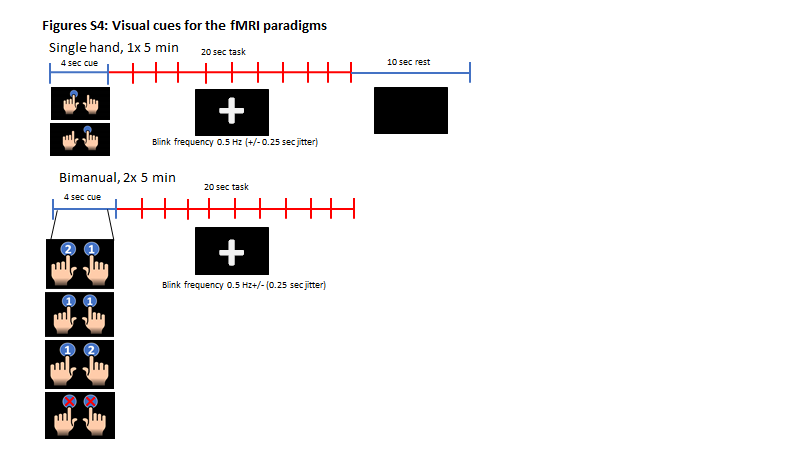

Supplement: Supplementary file 4 — Additional file 4: Figure S4. Visual cues for the fMRI paradigms. [file 13063_2023_7234_MOESM4_ESM.png]
